# Analyzing the specific role of cognitive functioning on successful aging

**DOI:** 10.1093/geroni/igab046.3665

**Published:** 2021-12-17

**Authors:** Neyda Ma Mendoza-Ruvalcaba, Elva Dolores Arias-Merino, Maria Elena Flores-Villavicencio, Maria Elena Rodriguez-Diaz

**Affiliations:** 1 Universidad de Guadalajara CUTONALA, Guadalajara, Jalisco, Mexico; 2 Universidad de Guadalajara, Zapopan, Jalisco, Jalisco, Mexico; 3 Universidad de Guadalajara, Universidad de Guadalajara, Jalisco, Mexico

## Abstract

Introduction The cognitive functioning, as a general measure, is a criterion commonly used to define and operationalize successful aging. (Project-Conacyt-256589) The aim of this study is to analyze specific domains of cognitive function and its relationship with the successful aging in older adults. Methods Population based, random sample included n=453 community-dwelling older adults 60-years and older (mean age=72.51,SD=8.11 years,59.4% women). Cognitive functioning was assessed by a comprehensive battery including working memory(Digit Span Backward WAIS-IV), episodic memory, meta-memory(self-report), processing speed(Symbol Digit WAIS-IV), attention(TMT-A), executive functioning(TMT-B), learning potential(RAVLT), language(FAS), visuospatial skills(Block Design WAIS-IV). Successful aging was operationalized as no important disease, no disability, physical functioning, cognitive functioning, and being actively engaged. Sociodemographic and health data were also asked. Data were analyzed in SPSSv24, MANOVAs and size effects were calculated. Results In total 11.2% were successful agers and 11.4% had Mild Cognitive impairment. Global cognitive functioning was significantly related to the achievement of successful aging criteria. Cognitive functioning had a significant effect on successful aging, specifically executive functions (F=1.07,p=.000) explained 32.7% of the variance, attention explained 29.8% (F=1.19,p=.006), processing-speed 21% (F=1.38,p=.000), and learning potential 21.5% ((F=1.12,p=.005). Language, visuospatial skills, working memory and meta-memory had a very small effect. Conclusion Knowledge generated by this study reveals the specific role of cognitive domains on successful aging, and sets a scenario to promote successful aging, through alternatives centered in the improvement of cognition in the older adults.

